# Advances in Methods and Technologies for Carcass and Meat Quality Evaluation

**DOI:** 10.3390/foods13172816

**Published:** 2024-09-05

**Authors:** Severiano Silva, Alfredo Teixeira

**Affiliations:** 1 Veterinary and Animal Research Centre (CECAV), Associate Laboratory for Animal and Veterinary Science (AL4AnimalS), University of Trás-os-Montes e Alto Douro, 5000-801 Vila Real, Portugal; ssilva@utad.pt; 2 Mountain Reserach Center (CIMO), Polytechnic Institut of Bragança, Campus de Santa Apolónia, 5300-253 Bragança, Portugal; 3 Laboratory for Sustainability and Technology in Mountain Regions, Polytechnic Institut of Bragança, Campus de Santa Apolónia, 5300-253 Bragança, Portugal

The importance of advanced methods and technologies in the meat industry has increased significantly in the last decade, reflecting broader trends in consumer demand and food safety. In this way, integrating advanced methods and technologies in the meat industry is not only meeting current challenges, but also paving the way for a more sustainable, efficient, and consumer-oriented future. As the industry evolves, these innovations will be critical in addressing global food security, health, and environmental issues supported by objective and reliable data. Hence, this Special Issue brings advances in different meat animal species and topics related to meat quality. Thus, articles were published to assess the meat characteristics of the Portuguese Bísaro pig breed; to characterize flavor volatiles in parts of Chinese Lueyang black chicken; the quality characteristics of the meat from dual-purpose poultry; the impact of dietary energy and protein levels on the quality and metabolomic profile of Yunshang black goats’ meat and the degradation of the intramuscular connective tissue with cathepsin L.

Using the Portuguese Bísaro pig breed, Vasconcelos et al. [1] evaluated the ability of the near-infrared reflectance spectroscopy (NIRS) coupled with partial least square (PLS) and support vector machine regression with the polynomial kernel (SVMR-Poly) models to estimate the water activity (aW), protein, moisture, ash, fat, collagen, texture, pigments, and water-holding capacity (WHC) of Longissimus thoracis et lumborum (LTL) muscle. The results show that, in general, the average root mean square error (RMSE), mean absolute error (MAE), and coefficient of determination (R2) results of the PLS and SVMR-Poly models are similar, with a slight quality advantage for the latter. Furthermore, it appears that PLS models require a high number of principal components (PCs), generally greater than 14, indicating that the models are complex, which implies a high number of variables. It is found that if the number of PCs is limited to 10, there is a decrease in the number of variables that can have predictive models, thus offering a more robust and reliable model to avoid overfitting, model interpretability, reduce computational complexity, and ensure better generalization. With this work, it was possible to conclude the potential of NIRS in determining the quality of Bísaro pork meat by obtaining suitable predictive models for the meat’s physical–chemical characteristics. The models that resulted from the SVMR-Poly approach, capable of modeling more complex, non-linear relationships between spectra and the physicochemical parameters, show that they are capable of being used in the area of the authentication and chemometrics of foods to prevent fraud and for the authenticity of products. This study demonstrates that NIRS has strong potential as an in situ analytical tool for meat quality assessment. Its key advantages include speed, low cost, acceptable levels of error, and that it does not require a specialized technician for operation. Consequently, NIRS presents a promising approach for classifying individual animals in breeding programs and for industrial applications to identify specific product breed characteristics.

He et al. [2] evaluated volatile organic components (VOCs) through gas chromatography–ion mobility spectroscopy (GC-IMS) combined with stoichiometry of six chilled chicken parts (breast, back, leg, heart, liver, and gizzard), in Lueyang black chicken. A total of 54 VOC peaks were detected using GC-IMS, with 43 VOCs identified through qualitative analysis. Aldehydes, ketones, and alcohols were the predominant categories. These included 22 aldehydes (20.7–54.1%), 8 ketones (25.7–62.9%), 9 alcohols (4.17–14.7%), 1 ether (0.18–2.22%), 2 esters (0.43–1.54%), and 1 furan (0.13–0.52%). Among the six parts, aldehydes had the highest relative content (54.1%) in the gizzard, ketones were most abundant in the heart (62.9%), and alcohols had the highest relative content (14.7%) in the liver. Principal component analysis (PCA) and clustering heatmaps showed that the characteristic VOCs effectively distinguished the six different parts of the Luoyang black chicken. In summary, GC-IMS combined with stoichiometry is a powerful tool to enable the detailed profiling of the volatile compounds that contribute to the chicken’s flavor, aroma, and potential health benefits, offering valuable insights for quality control, product development, and authenticity verification. As the demand for specialty poultry products like black chicken continues to rise, advanced analytical techniques like these will become increasingly important in ensuring product quality and consumer satisfaction. Furthermore, it is essential to reinforce the validity of the methods used in this study through future work involving a larger sample size.

Also related to poultry, Sharma et al. [3] studied the meat characteristics from a dual-purpose poultry crossbreed suitable for backyard rearing, which they compared with commercial broilers. This study examined the meat attributes of the Jabalpur Color (JBC), a dual-purpose, colored synthetic line developed by Nanaji Deshmukh Veterinary Science University, Jabalpur, India. The birds were raised under identical conditions in a deep litter system within a backyard, open-sided poultry house. They were sheltered at night and allowed to free-range during the day. The birds had unrestricted access to water and were fed ad libitum with a standard formulated diet. Male birds (n = 20/group) from the JBC chicken and the commercial Cobb 400 broiler were compared. At the age of marketing, the breast (pectoralis major muscle) and thigh (biceps femoris muscle) were dissected and used for biochemical analysis and gene expression proofing. The results indicate that the protein concentration in JBC breast meat (25.7 ± 0.39 g/100 g of tissue) and thigh meat (19.04 ± 0.23 g/100 g of tissue) was significantly higher (*p* < 0.05) than that of Cobb broilers. Established in vitro assays demonstrated significantly higher (*p* < 0.05) antioxidant capacity in JBC meat. High-performance liquid chromatography revealed substantial levels of functional biomolecules—carnosine, anserine, and creatine—in the JBC breast and thigh meat extracts. The average carnosine concentration (mg/g of tissue) was 2.66 ± 0.09 in the breast meat and 1.11 ± 0.04 in the thigh meat. The mRNA expression of carnosine-related genes—β-alanine transporter (SLC36A1), carnosine-synthesizing enzyme (CARNS1), and carnosine-degrading enzyme (CNDP2)—was quantified by qRT-PCR. Meat extracts from both genetic groups (JBC and Cobb) exhibited high anti-glycation potential. With this work, it was possible to conclude that JBC meat has superior nutritional and functional qualities compared to commercial chickens, with higher protein content and greater antioxidant capacity. These findings are not only beneficial for breeders, producers, and consumers, but they also empower consumers by bridging the knowledge gap between the meat quality of native chickens and the quantity yielded by commercial chickens.

Li et al. [4] studied the impact of dietary energy and protein levels on the meat quality and metabolic profile of goat meat using 80 Yunshang black male goats at six months old and a mean live body weight of 36 ± 2.8 kg. The experiment was conducted using a completely randomized design with a 2 × 2 factorial arrangement of diets. The dietary treatments were as follows: (1) high energy (9.74 MJ/kg) with high protein (12.99%) (HEHP), (2) high energy (9.76 MJ/kg) with low protein (10.01%) (HELP), (3) low energy (8.18 MJ/kg) with high protein (13.04%) (LEHP), and (4) low energy (8.14 MJ/kg) with low protein (10.05%) (LELP). The experiment spanned 64 days (14-day dietary adaptation and a 50-day feeding trial). After the experiment, four animals from each treatment group were slaughtered to evaluate their meat quality and metabolomic profiles. The pH value was highest in the goats fed the LELP diet compared to the other groups. Meat from the LEHP-fed group was significantly brighter (L*) than that from the other three groups. The HEHP-fed group produced more tender meat (*p* < 0.05) than the LEHP-fed group. Additionally, 72 and 183 differentiated metabolites were identified in the longissimus muscle samples using gas chromatography–mass spectrometry and liquid chromatography–tandem mass spectrometry, respectively. Significant differences (*p* < 0.05) in the hydropathy and volatilities of the raw meat were observed, indicating that the dietary treatments influenced meat flavor. The results suggest that a high-energy, high-protein diet enhances goat meat’s tenderness, flavor, and fatty acid content. In this study, a diet rich in energy and protein notably improved meat tenderness. The authors conclude that feeding strategies with varying dietary energy and protein levels can significantly alter goat meat quality and flavor. Further research on specific dietary energy and protein sources is needed to optimize meat quality.

Finally, the article by Peng et al. [5] expands knowledge to understand the factors linked to meat tenderness, a critical attribute for consumer satisfaction, which determines repeat purchases and the willingness to pay premium prices. This work has two objectives: (1) demonstrate that cathepsin L purified from bovine pancreas in vitro allows the degradation of collagen and decorin in intramuscular connective tissue (IMCT); (2) observe changes in the thermal transition of IMCT in the presence of cathepsin L. In this study, to obtain intramuscular connective tissue (IMCT), Longissimus lumborum (LL) samples were collected 36 h post mortem from the carcasses of 2.5-year-old cattle. The samples were subjected to several steps to purify cathepsin L from the bovine pancreas, including centrifugation, dialysis, and concentration. The concentrated samples were then sequentially processed through DEAE Sephacel anion exchange chromatography, Sephacryl S-100 HR size exclusion chromatography, and SP Sepharose FF cation exchange chromatography to remove large molecular weight heteroproteins, followed by A-Sepharose affinity chromatography. SDS-PAGE analysis of CNBr-digested peptides revealed that collagen degradation in intramuscular connective tissue (IMCT) likely occurs on terminal non-helical peptides rather than within the triple helix region. Decorin was notably degraded at a pH of 5.0. The onset (T_O_) and peak (T_P_) temperatures of thermal transitions in IMCT decreased to 41.41 °C and 43.79 °C, respectively. Under cathepsin L treatment at pH 5.0, the decreases in T_P_ and T_O_ were more pronounced than at pH 5.5–6.5. This study enhances our understanding of post mortem tenderization, providing theoretical support for developing new meat tenderizers.

## Data Availability

Data are contained within the articles.
